# Towards Fine Whole-Slide Skeletal Muscle Image Segmentation through Deep Hierarchically Connected Networks

**DOI:** 10.1155/2019/5191630

**Published:** 2019-06-27

**Authors:** Lei Cui, Jun Feng, Lin Yang

**Affiliations:** ^1^Department of Information Science and Technology, Northwest University, Xi'an, China; ^2^The College of Life Sciences, Northwest University, Xi'an, China

## Abstract

Automatic skeletal muscle image segmentation (MIS) is crucial in the diagnosis of muscle-related diseases. However, accurate methods often suffer from expensive computations, which are not scalable to large-scale, whole-slide muscle images. In this paper, we present a fast and accurate method to enable the more clinically meaningful whole-slide MIS. Leveraging on recently popular convolutional neural network (CNN), we train our network in an end-to-end manner so as to directly perform pixelwise classification. Our deep network is comprised of the encoder and decoder modules. The encoder module captures rich and hierarchical representations through a series of convolutional and max-pooling layers. Then, the multiple decoders utilize multilevel representations to perform multiscale predictions. The multiscale predictions are then combined together to generate a more robust dense segmentation as the network output. The decoder modules have independent loss function, which are jointly trained with a weighted loss function to address fine-grained pixelwise prediction. We also propose a two-stage transfer learning strategy to effectively train such deep network. Sufficient experiments on a challenging muscle image dataset demonstrate the significantly improved efficiency and accuracy of our method compared with recent state of the arts.

## 1. Introduction

Skeletal muscle accounts for approximately 40% body mass. As the largest body tissue, skeletal muscle has been extensively recognized as the biomedical health biomarker related to many diseases such as cancer cachexia, heart failure, and chronic obstructive pulmonary disease (COPD) [1–3]. In recent years, the growing attentions, in the muscle biology community, have been paid to the analysis of histological images of skeletal muscle to assist the diagnosis of relevant diseases [1].

The quantification of morphological characteristics of muscle fibres plays an important role in the assistance of disease diagnosis and clinical studies. Critical morphological characteristics, including cross-section area, fiber type and shape, and the minimum Feret diameter, are closely related to the functionality and health of muscle [4, 5]. To accurately quantify these morphological characteristics of muscle fibres, an accurate skeletal muscle image segmentation (MIS) system is the prerequisite.

Currently, the segmentation for muscle fibres in routine practice still highly relies on experts' manual labors or semiautomatic process [6], which are not only expensive but also contain large interobserver variations. The increasing demand of a fast and accurate automatic MIS attracts many attentions recently. Various approaches have been proposed to address this task [4, 7–9].

The difference between MIS and standard histological image cell segmentation is attributed to the specific morphology of skeletal muscle. Skeletal muscle is composed of long, multinucleated cells (fibres) tightly grouped into fascicles, interspersed with other mononucleated cell types and surrounded by connective tissues and fat. This tightly grouped anatomical structure, coupled with artifacts and staining variances introduced during sample preparation, generates confusing and overlapping cell boundaries.

Although histological cell segmentation research has a rich history, few have been successfully applied to MIS. There are still several challenges which remain to be solved to achieve a robust and automatic MIS system. First, all exiting MIS methods can only handle small image patches with the size smaller than 1000 × 1000 cropped from whole-slide muscle images. The main reason is that supervised methods usually rely on handcrafted features and pretrain classifiers to distinguish cell or noncell regions or pixels. However, the computation of well-designed handcrafted features and regionwise classification is usually expensive and the time cost is proportional to the image scale. Therefore, this limitation of existing methods makes them hardly be applied for large-scale, whole-slide muscle images.

Second, the special muscle cell shape and size as abovementioned make the segmentation methods hardly be generalized. For example, unsupervised methods, such as the deformable model [10, 11] and shape prior-based methods [12, 13], have been widely used in histological and microscopy cell segmentation. However, the arbitrarily transformed cell shapes and size increase the difficulty to use shape information for MIS.

Third, the densely touching fibres and staining artifacts make the fiber boundaries unclear and broken, which increases the difficulties for methods to separate multiple touching fibres by using boundary information. Fourth, current methods usually contain multiple mutually dependent steps; the failure of either step will largely affect other steps and the final results. On the other hand, the complex pipeline largely decreases the speed of MIS.

This paper addresses these challenges to achieve a both efficient and effective MIS method based on recently popular convolutional neural network (CNN). CNN-based methods have achieved unprecedented performance in various medical image applications. Different from conventional computer vision methods, CNN has strong capability to learn comprehensive representations via a deep architecture for effective classification. When using CNN for pixelwise classification, conventional CNN shows the efficiency shortcomings [14]. The end-to-end CNN training strategy has recently attracted a lot of research interest [15, 16]. However, a common problem is that the dense output is relatively coarse, and it is difficult to accurately classify each pixel [16, 17]. To generate more accurate and fine outputs, a refinement procedure needs to be considered.

**Figure 1:**
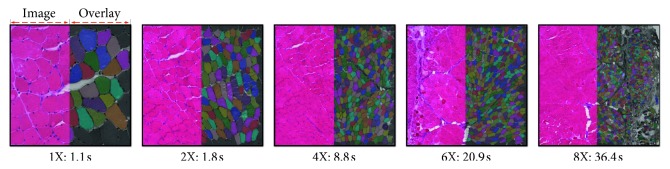
Illustration of the segmentation results of different scale (1x=1000 × 1000 pixels to 8x=8000 × 8000 pixels) whole-slide muscle images (best viewed in electronic form). For each image, the right half side represents the segmentation results overlaid by the colored masks. The runtime is the result tested on a single GPU.

**Figure 2:**
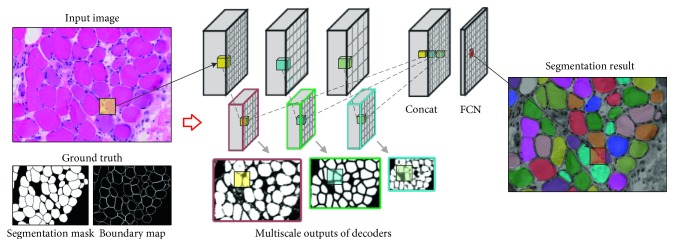
The illustration of the network architecture. The input image has a ground truth segmentation mask and a boundary map. Black boxes indicate the encoder module while colored boxes indicate the decoder module. One decoder takes the feature maps of one encoder layer as input and outputs one segmentation results. The multiscale outputs of all encoders are concatenated to generate the final segmentation result.

In this paper, we propose a novel MIS method based on CNN trained in an end-to-end manner [16], which enables the CNN to better utilize the rich representations and directly predict fine-grained segmentation given an arbitrarily sized input image.  Figure 1 shows some segmentation results of different image scales. Specifically, the main contributions of this paper are summarized as follows:

- We propose a network whose architecture mainly contains two modules: the encoder and the decoder. The encoder captures rich representations through a very deep CNN architecture. The decoder leverages on the hierarchy characteristic of the encoder to enable multiscale prediction independently. A refinement procedure of the decoder automatically addresses the fine-grained dense outputs.  Figure 2 illustrates our network.
- We propose a novel spatially weighted loss function to take care of the unbalanced class issue and unavoidable errors happened in ground truth, which encourage the convergence of the network.
- We propose a two-stage training approach to train the proposed very deep network, which facilitates the network to better use pretrained CNN for better convergence and preserve the weak boundary information of muscle cells.
- We conduct sufficient experiments on an expertise-annotated skeletal muscle image dataset demonstrating the significantly improved efficiency and accuracy compared with other state of the arts.

## 2. Related Works

The growing interest in the computer-aided histological image diagnosis entails rich research literature. As one of the histological image analysis family, skeletal muscle image analysis is a new yet recently popular application which has built successful cooperations with clinics to accelerate their research and clinical trials [1–3, 18, 19].

As the prerequisite of skeletal muscle image analysis, various methods have been proposed for MIS. Klemencic et al. [10] proposed a semiautomatic muscle image segmentation approach based on the active contour model. Janssens et al. [8] proposed a top-down cell segmentation framework using supervised learning and clump splitting, which requires a long pipeline with the help of several low-level image processing techniques. However, the performance of these image processing techniques can be easily influenced by imaging artifacts and cell clumps. Smith and Barton [6] proposed SMASH—semiautomatic muscle image analysis software. Some other software applications such as CellProfiler [9] have obtained high exposure in histological image analysis community. However, these software applications show nonsatisfactory results for challenging muscle images. Practically, time-consuming manual adjustment is still needed. Liu et al. [4] proposed a deformable model-based segmentation algorithm. The success relays largely on the initial centers of the muscle cells. It is not able to handle cells with arbitrarily transformed fiber shape, and it requires complex postprocessing to refine the results, which is not robust in practice. Recently, Liu et al. [7] proposed a hierarchical tree-based region selection method to segment muscle fibres, which relies on elaborately designed features and high-level machine learning techniques. This method first detects fiber boundaries by using structured random forest [20]; then, it builds a hierarchical region tree based on the detected edge map. Finally, the dynamic programming is performed to select candidate regions from the tree structure [21]. This method shows obvious improvement compared with previous MIS approaches. However, this method still suffers from relatively expensive computation, so it is unable to be applied onto whole-slide images.

As a matter of fact, whole-slide MIS is still an unsolved problem. Although some literature discusses the usage of distributed computing [22–24] to accelerate process for large-scale histological images, distributed computing is usually difficult to be deployed for practical usage in clinical practice.

Convolutional neural network (CNN) [25] is one major branch of the deep learning family. Its applications in pathology and histological image analysis domain became increasingly popular very recently [14, 26–28]. CNN has shown strong ability to handle complex classification problem [29]. Recently, end-to-end CNN training concept is introduced for semantic image segmentation, termed fully convolutional neural network (FCN) [16]. Instead of performing patch-to-pixel prediction, it enables the network to perform spatial dense classification (i.e., a segmentation mask) given a test image. By taking advantaging of this strength, several methods have been proposed to handle various pixelwise classification tasks [15, 30–34]. Our paper shares some similarity with the previous works of how to enable CNN to be trained in an end-to-end manner. Different from previous works, we have made several specific designs to handle fine-grained prediction, unbalanced class, multiscale features, and transfer learning from pretrained model for MIS. More details are discussed in the rest of the paper.

## 3. Methodology

In this section, we begin by introducing the proposed network architecture and then present proposed loss function for training the network. Finally, we introduce the two-stage learning to train the overall network.

### 3.1. Network Architecture

We briefly introduce the convolutional neural network (CNN) at first. CNN [25] is a variant of multilayer perception (MLP), which is mainly composed of multiple stacked computation layers from bottom to top, including convolutional, max-pooling and fully connected layers, activation layer, etc. The convolutional layer uses learnable convolutional filters to extract representations from locally connected image regions (receptive fields). The max-pooling layer reduces the dimensionality of the obtained representations from convolutional layers while keeping the feature translation invariance. The fully connected layer uses all features for high-level classification. From bottom layers to top layers, CNN gradually captures rich representations of input image from pixel level to content level so as to make accurate classification. Conventional CNN is used to perform high-level classification, i.e., assigning a category label to an input image patch. When it is applied to pixelwise prediction MIS task, extensive patch-to-pixel level prediction (CNN feedforward) is required, which will extensively limit the segmentation efficiency [14, 35].

To solve this problem, we train our network in an end-to-end manner, which enables the network to directly output the image segmentation given an input image. In this way, we no longer need to use patchwise classification to assign labels to all pixels via millions of CNN feedforward. Only one-time feedforward is needed to obtain the final segmentation. End-to-end training of CNN is used to enable the network to directly output dense image segmentation for a given input image [15, 16, 32].

However, modifying conventional CNN to perform end-to-end training brings a major side effect, i.e., substantial pixel-level information loss at top layers makes the pixelwise prediction inaccurate [17, 30]. It is because multiple max-pooling layers will dramatically decrease the spatial size of the output, so the predicted segmentation output is very coarse. Most proposed end-to-end CNN methods use upsampling [16, 17] or deconvolution operations [15] to resize back the output to the spatial size of the input image. Nevertheless, the max-pooling layer is essential to abstract the content-level representations for high-level category classification [25, 29, 36] and decrease the computation space of CNN.

As a matter of fact, when we generalize end-to-end CNN to MIS, content-level information becomes less important because the label of a single pixel does not rely on the knowledge of the whole muscle image. Different from semantic segmentation [16, 32] which needs content-level information to predict the category label per pixel, we are more interested in the fine-grained pixelwise prediction by taking advantage of the hierarchical representations of the encoder to improve the prediction accuracy. The hierarchy characteristic can be achieved by gradually enlarging the receptive field size after each max-pooling layer. To this end, we propose a novel network architecture, which is composed by one encoder module and multiple decoder modules. Generally, the decoder aims to use the rich and hierarchical representations obtained from the encoder for pixelwise classification.

#### 3.1.1. Encoder Module

The encoder architecture is mostly identical to the conventional neural network. Instead of building our own layer combinations, we borrow the well-known VGG net [29] with fully connected layers truncated to capture the rich and hierarchical representations from pixel level at bottom layers to content level (i.e., category-specific knowledge) at top layers. VGG net is composed of a series of convolutional sets with each set having multiple convolutional layers followed by a max-pooling layer. VGG has two variants (one has 16 layers and the other has 19 layers); we use 16-layer VGG for efficiency consideration. We choose VGG for two reasons: (1) we can transfer the pretrained VGG model to help train our very deep network as described in the next section; (2) VGG net is very deep which extracts five different-scale feature maps, containing very rich multiscale representations for the usage of decoders.

#### 3.1.2. Decoder Module

The decoder has two main purposes: (1) it utilizes the rich representations obtained from the encoder for pixelwise classification. So, the output of one decoder is a dense segmentation mask with each spatial position assigning a label to the corresponding pixel of the input image (cell or noncell in our case); (2) it refines the low-rescale coarse segmentation mask to efficiently generate fine-grained high-scale segmentation mask. The refinement procedure is achieved by multistep deconvolution and successive usage of same-scale feature maps obtained from other decoders.

We propose to connect multiple decoders prior to every max-pooling layer of the encoder; thus, the decoders can easily utilize the multiscale representations as input features as inspired by [15, 16, 31]. The decoder can be viewed as a small pixelwise classification network, which has an independent loss to update its parameters during training. Hence, the overall architecture is multitask CNN.

Our design of the decoder includes convolutional layers with intermediate deconvolution layers [15]. Specifically, the deconvolution is the backward convolution operation, which performs elementwise product with its filters (please note that some controversies arise in the naming of “deconvolution” in recent literature as the deconvolution layer used here is different from the previous definition of the deconvolution [37]; we maintain the same definitions as most of the literatures on end-to-end CNN). The output size of deconvolution will be enlarged by a factor of the stride. The filters of the deconvolution layers are learnable and updated by the loss of the decoder.

**Figure 3:**
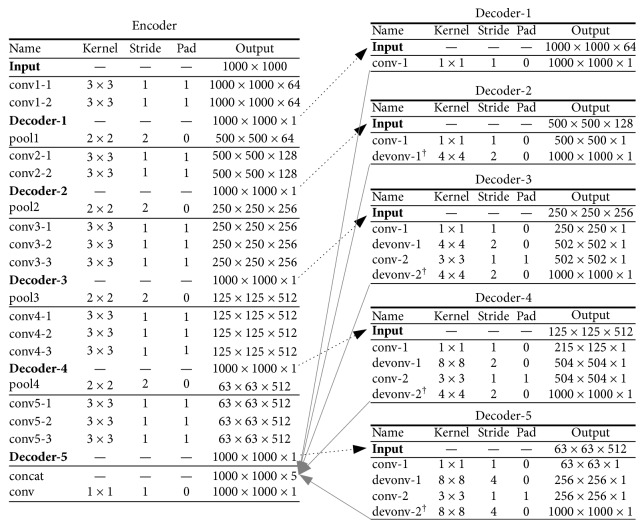
The detailed network configuration. The convolutional, max-pooling, deconvolutional, and concat layers are denoted by conv, pool, deconv, and concat, respectively. Each convolutional layer of the encoder is followed by a ReLU layer which is hidden in the tables. There are 5 decoders connected inside the architecture of the encoder. The (black solid and gray dotted) arrows point to the layer where the output of the corresponding layer goes. The last column of each table shows the feature map size (height ×  width ×  dimension) of each layer. In the tables of decoders, “†” indicates that a crop layer is connected after that to force the output size to be the same as the input image size (i.e., 1000 × 1000 in the table).

In this way, rather than enlarging the image with a large stride through a skip connection [16, 31, 38], our approach enlarges the feature map in multiple steps and progressively refines the feature maps at different scales via convolutional kernels, with the purpose of reducing the effects of pixel-level information loss. We use 3 × 3 filter size as this small size has been proven effective widely. In the end, we concatenate multiscale predictions of all decoders, which generates a 5-dimensional feature map; we apply a 1 × 1 convolutional layer to merge the feature map to generate the final output. Compared with how recent architecture [35, 39] uses multiscale information (resize input patch size and feed into multiple CNNs and merge all predictions [35, 39]), our approach enables multiscaling inside the network, requiring a single arbitrarily sized input and outputting the final segmentation result.  Figure 3 specifies the parameters of each layer.

### 3.2. Spatially Weighted Loss for Backpropagation

This section describes the loss function training network through backpropagation. Our proposed spatially weighted loss plays an important role in network training.

Denote the training data as *𝒟*={(*X*, *Y*) ∈ *𝒳* × *𝒴*}, where *𝒳*, *𝒴* ∈ *ℝ*^*N*^ and *N* is the total number of pixels in the training image *X*. *Y* is the corresponding ground truth segmentation mask with each pixel *Y*_*i*_ ∈ {0,1} (i.e., pixels inside and on the boundary the muscle cell and background otherwise). For an input image *X*, the main objective of our network is to obtain the pixelwise prediction *Y*^*⋆*^:

(1) $$\begin{array}{c} Y^{⋆}=\underset{\hat{Y}}{\arg\;\;\max}P\left(\hat{Y}\;|\;X,\;\;\theta \right), \end{array}$$

where $P\left({\hat{Y}}_{i}|X;\theta \right)$ is the prediction probability of pixel *X*_*i*_, i.e., the *sigmoid* function output of the network (denoted as *P*_*i*_ afterwards for brevity). *θ* represents all parameters of our network.

Our network has multiple decoders with each having independent loss to update their parameters (see Section 3.1.2 for details). Denote the loss function of *i*-th decoder as *𝒥*_*i*_^de^. The extra 1 × 1 convolutional layer after the concat layer is updated by another loss (see Figure 3), denoted as *𝒥*^*c*^. Learning *θ* is achieved by minimizing the loss function *𝒥*, which is defined as

(2) $$\begin{array}{c} J\left(\theta \right)=\overset{M}{\underset{i=1}{\sum }}J_{i}^{\text{de}}\left(\theta \right)+J^{c}\left(\theta \right), \end{array}$$

where *M* is the number of the decoder. Note that since *𝒥*_*i*_^de^ and *𝒥*^*c*^ are both spatially computed on pixels of the dense output, both have the same formulation. The overall loss *𝒥* can be jointly minimized via backpropagation (specifically, when a layer has more than one path, such as the conv1-2 layer in Figure 3 which has two successive layers (decoder-1 and pool1), the gradients will be accumulated from multiple successive paths during backpropagation [40]).

In skeletal muscle images, there are several common problems which affect the network training: (1) the large proportion of pixels inside cell pixels will cause an unbalanced class such that the error effects occurred at the margins will be diminished during backpropagation; (2) usually cells are densely distributed and the boundaries between touching cells are thin and often unclear or broken due to muscle's unique anatomy; based on our observations, the network often misclassifies the pixels at margins between fiber boundaries; (3) due to the staining issue, the boundary pixels are not smooth and continuous, so it is very difficult to ensure that annotations accurately label each pixel. It is necessary to reduce the ambiguity for network training.

We propose a loss function to ameliorate these problems by assigning different weights to the loss of each pixel. The loss function of a training data *X*, which is based on the cross-entropy loss, is defined as

(3) $$\begin{array}{c} J^{\text{de}}\left(\theta \right)=\overset{N}{\underset{i=1}{\sum }}f\left(X_{i}\right)\left(1\left[Y_{i}=1\right]\log\;P_{i}+1\left[Y_{i}=0\right]\log\left(1-P_{i}\right)\right). \end{array}$$

The pixelwise weights are defined by the weight-assigning function *f*, which is defined as

(4) $$\begin{array}{c} f\left(X_{i}\right)=C{\left(Y_{i}\right)}^{-1}\times \;\;\exp\frac{\Omega \left(X_{i}\right)}{\eta_{1}}\times 1\left[\left|Y_{i}-P_{i}\right|<\eta_{2}\right]. \end{array}$$

The pixelwise weight-assigning function *f* has three terms, which play different roles to address the abovementioned three problems. The specific considerations make our proposed loss different from [16, 30].

In the first term, *C*(*Y*_*i*_) is the label frequency, which is a global term having the same value for same-class pixels. In second term, *Ω* is the Euclidean distance of pixel *X*_*i*_ to the boundary of the close cell. Similar to [30], the intention of *f* is to assign relatively high weights to pixels adjacent to boundaries to amplify the error penalty occurred at the margins and pixels close to fiber boundaries and 1 otherwise (*Ω*=0 if *Ω*(*X*_*i*_) > *ε*). We set *η*=0.6 and *ε*=10 empirically. Compared with the “hard” error-balancing strategy in [16, 31], *f* produces soft error penalty so as to encourage better optimization convergence and enhance fine-grained prediction. The third term aims to reduce the reliability of the ground truth when the network predicts an opposite label with high probability. This term is a switch, so it forces the weight of the corresponding pixel to zero when the condition is not satisfied. In practice, we preserve this value during network feedforward, while the loss of the corresponding pixels does not get involved during network backpropagation.

### 3.3. Two-Stage Training

Training our deep network has some common difficulties:

- The large number of parameters in both convolutional layers and deconvolutional layers makes the training difficult to achieve proper convergence [15, 41].
- Successful training from scratch requires extensive labeled data which are extremely difficult to obtain in medical image domain.

One typical solution is to apply transfer learning to reduce the training difficulty [41, 42], which reduces the difficulties of the tricky parameter initialization and tuning [25, 29] and heavy data acquirement procedure. The core idea behind is to use a pretrained model as the initialization and fine-tune the CNN to make it adapt to targeting tasks with new training data. The encoder of our network partially inherits the architecture of VGG [29], which is, however, trained on a large set of natural images for image classification. Transferring its knowledge to benefit the totally unrelated biological image analysis problem (i.e., MIS) seems impracticable. However, a recent literature coincides with our experiments. It demonstrates the advantage [41] using various biological imaging modalities transferring from AlexNet [25], a relatively shallow CNN for natural image classification. In terms of our MIS case segmentation, the network architecture is much more deeper with many new parameterized layers in decoders. More specific treatment needs to be considered.

It is well known that the bottom layers of CNN can be understood as various feature extractors attempting to capture the low-level image features such as edges and corners [25, 37, 41]. Actually, those low-level features are common between natural images and muscle images, of which the most common feature is image gradients (i.e., boundaries). In practice, we find that training the network to detect boundaries is relatively easier than directly training the network to segment muscle fibres.

We propose a two-stage training strategy to progressively train our network so as to utilize the powerful feature extractors of VGG and overcome the abovementioned problems. In the first stage, we apply transfer learning to use pretrained VGG to initialize the parameters of the encoder and randomly initialize the parameters of decoders. We then train the network to detect fiber boundaries, which is achieved by feeding the network with training muscle images associated with the ground truth boundary map (see Figure 2). This strategy will facilitate the network to converge swiftly. After the network becomes adapted to new muscle images, in the second stage, we fine-tune the model using the original training data *𝒟* (i.e., *Y* is the segmentation mask) to train the network to automatically segment muscle fibres, assigning in-cell pixels to 1 and other pixels to 0. More implementation details are described in the experimental section.

Another advantage of our proposed training strategy is that it further helps reduce the touch objects (due to thin boundaries) problem [30, 34] commonly occurred in end-to-end CNN segmentation (besides the pixel weight-assigning function *f*). The strategy of this literature [34] is to predict both a segmentation map and boundary map and merge two maps to solve touching glands. While in our method, the first stage training makes the network detect the cell boundaries. The second stage training is able to preserve this boundary information.

## 4. Experimental Results

### 4.1. Dataset

Our expert annotated skeletal muscle image dataset with H&E staining contains 500 annotated images, which are captured by the whole-slide digital scanner from the cooperative institution Muscle Miner. The images exhibit large appearance variances in color, fiber size, and shape. The image size roughly ranges from 500 × 500 to 1500 × 1500 pixels. We split the dataset into 100 testing images and 400 training images.

In order to evaluate the proposed method to handle large-scale images, we evaluate the runtime on a whole-slide image. Note that we use small image patches for segmentation accuracy evaluation because some comparative methods in the literature cannot handle whole-slide images. However, our proposed network is flexible to the input size during the testing stage because the decoder is able to adaptively adjust the output size to be consistent with the input size.

### 4.2. Implementation Details

Our implementation is based on the Caffe [40] framework with modifications for our network design. All experiments are conducted on a standard desktop with an Intel i7 processor and a single Tesla K40c GPU. The optimization is driven by stochastic gradient descent with momentum. For the first stage training, the network parameters are set to learning rate =1*e* − 6 (divided by 10 every 1*e*4 iteration), momentum =0.9, and minibatch size =2. In the second stage, we use the learning rate =1*e* − 7 and keep the others the same.

Augmenting dataset is a normal step for training CNN. We apply a simple approach by randomly cropping 30 300 × 300 image patches from each of the training images to generate totally 1.2*e*4 training data. We choose this patch size to take the memory capacity of GPU into account. Based on our observations, the segmentation accuracy will not be affected by increasing input size of test images. To simplify the computation of the weighting function *f* during training, we take another per-computed weighting map associated with each training data (*X*, *Y*) as network inputs.

### 4.3. Segmentation Accuracy Evaluation

For quantitative evaluation, we report Precision =(|*S* ∩ *G*|/|*S*|), Recall =(|*S* ∩ *G*|/|*G*|), and *F*_1_-score =(2 · Prec.·Rec./Prec.+Rec.), where |*S*| is the segmented cell region area and |*G*| is the corresponding ground truth region area. For each test image, Precision and Recall are computed by averaging the results of all fibres inside. We report the three values with a fixed threshold (FT), i.e., a common threshold produces the best *F*_1_-score over the test set, and dynamic thresholds (DT) produce the best *F*_1_-score per image.

In Table 1, we compare the segmentation performance of our approach to several state-of-the-art methods. DC [43] and multiscale combinatorial grouping (MCG) [44] are recently proposed learning-based image segmentation methods. U-Net [30] is an end-to-end CNN for biomedical image segmentation. We use their public codes and carefully train the models over our training data with the same amount. DNN-SNM [14] is a well-known CNN-based image segmentation method. We regard it as a generic CNN for comparison with our end-to-end CNN approach. For our method, we directly use the network output as the segmentation results for evaluation without any extra postprocessing efforts.

**Figure 4:**
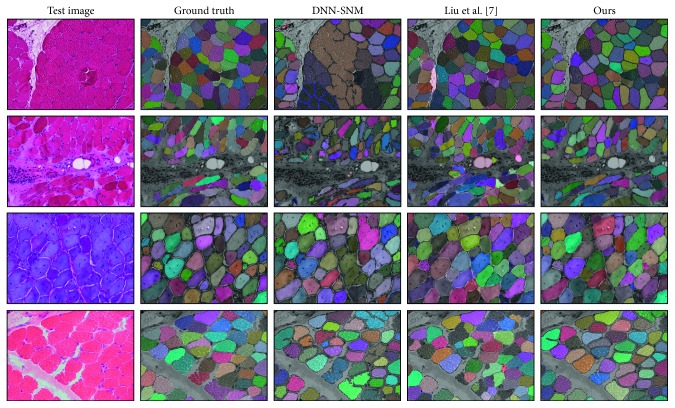
Segmentation results of four sample skeletal muscle images. We show some very challenging cases with large appearance variances in color, fiber shape, etc. Each segmented fiber is overlaid with a distinctive colored mask while false positives and false negatives are highlighted by red and blue contours, respectively. Compared with the other two methods, our method obtains more fine-grained segmentation results with obviously less false prediction.

As shown in Table 1, our method achieves much better results than comparative methods. Although [7] has better Recall (FT), our method has around 10% improvement on Precision (FT). DC and MCG are not robust to the image artifacts, which decreases their segmentation performance. Our method largely outperforms DNN-SNM and U-Net because (1) our network is deeper than DNN-SNM to capture richer representations, (2) the decoder better utilizes the multiscale representations than U-Net and is able to reduce the effects of the pixelwise information loss, and (3) two-stage training takes advantage of VGG for better training effectiveness rather than training from scratch as U-Net does. The outstanding Precision result demonstrates that our method produces more fine-grained segmentation than others. This superiority is better demonstrated by the qualitative evaluation as shown in Figure 4.

### 4.4. Whole-Slide Segmentation Runtime

In Table 2, we compare the runtime of our method to the comparative methods on images of different sizes cropped from a whole-slide image (see Figure 1). The runtime of non-deep learning-based methods (1st block) depends on both pixel and fiber quantities, so they cannot handle large-scale images. In contrast, deep learning-based methods (2nd and 3rd blocks) depend on the pixel quantity, so they have close-to-linear time complexity with respect to the image scale. We also implement a fast scanning version [45] of DNN-SNM on GPU. Although the speed has a large improvement, it is still much slower than ours. U-Net has more complicated layer connection configuration, so it is slower than ours, especially in large-scale cases. The significant speed improvement demonstrates the scalability of our proposed method to the application of whole-slide MIS with even larger scales.

## 5. Conclusion

This paper presents a fast and accurate whole-slide MIS method based on CNN trained in the end-to-end manner. Our proposed network captures hierarchical and comprehensive representations to support multiscale pixelwise predictions inside the network. A two-stage transfer learning strategy is proposed to train such a deep network. Superior accuracy and efficiency are experimentally demonstrated on a challenging skeletal muscle image dataset. In general, our approach enables multiscaling inside the network, while just requiring a single arbitrarily sized input and outputting fine outputs. However, during the downsampling process of the encoding, due to the limitation of resolution of feature layer after downsampling, many important features, such as edge features of cells, are still lost. To further improve decoding efficiency, in the future work, we can design a module that complements important features to better improve network performance.

## Figures and Tables

**Table 1:** The segmentation results compared with state-of-the-art methods.

Method	*F*_1_-score (%±*σ*)		Precision (%±*σ*)		Recall (%±*σ*)	
	FT	DT	FT	DT	FT	DT
DC [43]	48 ± 0.093	60 ± 0.138	41 ± 0.066	54 ± 0.164	67 ± 0.194	73 ± 0.148
MCG [44]	63 ± 0.201	71 ± 0.105	53 ± 0.136	64 ± 0.138	80 ± 0.303	82 ± 0.091
DNN-SNM [14]	76 ± 0.033	78 ± 0.080	83 ± 0.042	85 ± 0.089	70 ± 0.058	73 ± 0.087
U-Net [30]	80 ± 0.143	81 ± 0.054	87 ± 0.155	86 ± 0.076	74 ± 0.126	77 ± 0.055
Liu et al. [7]	82 ± 0.172	84 ± 0.061	81 ± 0.043	84 ± 0.071	**85** ± **0.202**	85 ± 0.068
Our approach	**86** ± **0.184**	**89** ± **0.048**	**91** ± **0.174**	**93** ± **0.050**	82 ± 0.176	**86** ± **0.058**

*σ* is the standard deviation.

**Table 2:** The runtime (in seconds) comparison on images of different sizes from 1x=1000 × 1000 to 9x=9000 × 9000.

Method	1x	2x	3x	4x	5x	6x	7x	8x	9x
DC [43]	20	79	—	—	—	—	—	—	—
MCG [44]	7	27	—	—	—	—	—	—	—
Liu et al. [7]	10	59	—	—	—	—	—	—	—
DNN-SNM [14]	264	1056	2376	4224	6600	9504	12936	16896	21384
DNN-SNM^*⋆*^[45]	31	115	242	431	675	974	1325	1738	2160
U-net [30]	1.2	3.9	9.0	16.1	24.6	36.8	48.2	63.3	79.2
Our approach	**1.1**	**1.8**	**5.3**	**8.8**	**13.9**	**20.9**	**27.8**	**36.4**	**46.8**

The first three methods cannot handle images with 3x and larger sizes on our machine (represented with “—” in the table). ^*⋆*^DNN-SNM is a fast scanning implementation for prediction speed acceleration.

## Data Availability

The data that support the findings of this study are available from the cooperative institution Muscle Miner, but restrictions apply to the availability of these data, which were used under license for the current study and so are not publicly available. Data are however available from the authors upon reasonable request and with permission of the cooperative institution Muscle Miner.
